# Quality of referrals to specialist palliative care and remote patient triage — a cross-sectional study

**DOI:** 10.1007/s00520-023-08025-6

**Published:** 2023-09-02

**Authors:** Tomasz Grądalski, Krystyna Kochan

**Affiliations:** 1 Chair of Palliative Medicine, Andrzej Frycz Modrzewski Krakow University, Kraków, Poland; 2 St. Lazarus Hospice, Fatimska 17, 31-831 Kraków, Poland

**Keywords:** Eligibility determination, Palliative care, Referral, Remote consultation, Triage

## Abstract

**Purpose:**

Choosing the optimal moment for admission to palliative care remains a serious challenge, as it requires a systematic identification of persons with supportive care needs. Despite the screening tools available for referring physicians, revealing the essential information for preliminary admission triage is crucial for an undisturbed qualification process. The study was aimed at analysing the eligibility criteria for specialist palliative care disclosed within provided referrals, expanded when necessary by documentation and/or interview.

**Methods:**

Referral forms with the documentation of 300 patients consecutively referred to the non-profit in-patient ward and home-care team in Poland were analysed in light of prognosis, phase of the disease and supportive needs.

**Results:**

Half of the referrals had the sufficient information to make a justified preliminary qualification based solely on the delivered documentation. The majority lacked performance status or expected prognosis. Where some information was revealed, two-thirds were in a progressing phase of the disease, with a within-weeks life prognosis. In 53.7%, no particular reason for admission was given. Social problems were signalled as the only reason for the admission in 7.7%. Twenty-eight percent were labelled as “urgent”; however, 52.4% of them were triaged as “stable” or disqualified. Patients referred to a hospice ward received complete referral forms more often, containing all necessary information.

**Conclusions:**

General physicians need practical tips to facilitate timely referrals and unburden the overloaded specialist palliative care. Dedicated referral forms extended by a checklist of typical patients’ concerns should be disseminated for better use of these resources.

**Supplementary Information:**

The online version contains supplementary material available at 10.1007/s00520-023-08025-6.

## Introduction

Palliative care is focused on the relief of suffering due to severe illness, particularly, near the end of life. However, patients being at earlier phases of the disease are also indicated [1]. In Poland, three types of such care are refundable: consultations in outpatient clinics and continued care at homes or inpatient wards. The national health care reimbursement system does not distinguish palliative from hospice care and restricts it to mainly advanced cancer patients. Apart from that, there are no well-defined medical criteria for admission. Moreover, some persons can be legally referred both to fully reimbursed palliative and partially paid long-term facilities. In practice, a substantial number of patients referred from oncological centres or hospitals to palliative care bypasses the activity of general practitioners. As a consequence, a growing number of people continue to swell the waiting lists, some of them dying before admission [2]. Due to a lack of precise national legislation, palliative care facilities are trying to create their own admission systems, depending on individual caseload (in terms of patients with advanced cancer being referred) or institutional funding opportunities. The screening process of patients’ needs performed by palliative institutions is not infrequently delayed until the day of admission. This situation calls for urgent activation of the palliative approach within the entire health care system [3].

In a recent systematic review of palliative needs screening tools, it has been indicated that most of them use either prediction of death or deterioration, or both, as proxies for the identification of people who have such unmet requirements [4]. Two main groups of clinical referral criteria were distinguished for patients with life-limiting or end-stage diseases: prognosis- and needs-based: severe, complex or persistent, especially at the dying phase and the need for specialised dynamic support due to a spiritual or existential crisis [5]. Although there are numerous observations regarding the positive impact of reducing unnecessary hospitalisations near end of life on its quality, it is difficult to establish a consensus on characteristics of the optimal time for referral. Despite the fact that attempts of international experts according to the outpatient palliative care were made [6], many discrepancies remain, particularly in non-cancer diseases, such as advanced heart failure or dementia [7, 8]. Meanwhile, palliative care, compared to that commonly applied also in non-cancer patients, was associated with less acute health care use and a modestly lower burden of symptoms [9].

Choosing the optimal moment for referral to inpatient and home-based palliative care remains a serious challenge in most countries, as it requires a systematic process to identify persons with high supportive care needs [10]. Such a personalised process of referrals may provide a more rational use of the scarce resources, while maximizing their impact. Timely referral largely depends on physicians’ (mainly oncologists, general practitioners and hospital doctors) beliefs, assumptions, training, and competence regarding whether they have needs that require the involvement of specialist palliative care [11, 12]. The convictions of these physicians and a detailed description of supportive needs among the referred patients remain scarce. This study was aimed at exploring the overall documented reasons and needs for palliative referral within referral forms.

## Patients and methods

Referral forms, with attached documentation of all consecutive patients referred to the palliative in-patient ward at the non-profit, free standing hospice and palliative home-care team, localised in a metropolitan city of 780 thousand inhabitants in Poland, between 21 September and 1 December 2022, were analysed and collated with the preliminary qualification. The process of qualification performed routinely by the authors, immediately after registration, was based on referral content, supplied documentation and, when necessary to make a justified decision, additional telephone interview. The completeness of the referral was made based on the presence of information concerning disease stage, patient’s performance, symptoms of suffering, previously applied therapies and social concerns, which were covered by the dedicated referral form available on the hospice web site (Supplementary file). Triage as “urgent case” (in opposition to “stable” one) was made when the patient had severe (based on the Edmonton Symptom Assessment System six-point Verbal Rating Scale [13]) or complex (multiple) signs or symptoms, which were resistant to previous medical attempts of management. Referrals were not accepted when patients did not have symptoms or signs of at least moderate intensity, were in the stable phase of the disease or had a diagnosis which was non-refundable. In the case of such disqualification, adjusted information was given to the patient or his/her family, and a consultation within the palliative medicine outpatient clinic was advised.

We summarised the data using descriptive statistics, including counts and percentages. The Mann–Whitney test was performed to assess categorical variables of age and the number of noted problems. McNemar’s test was conducted to compare the completeness of referral forms, while the Chi square test was implemented to determine if there were differences in categorical variables within the subgroups. Spearman’s signed rank correlation test was applied to measure the relationship between the number of problems reported and referral triage outcome. A *p* value of 0.05 was considered statistically significant. The data were coded before the analysis to ensure anonymity of the participants. Institutional ethics committee approval was obtained for this prospective observational study.

## Results

Three hundred patients (94% with cancer), 150 being referred to the hospice ward, were assessed (162 men; 54.0%); the mean age being 74 years (interquartile range, IQR 64.0–82.0). The women were significantly older than the men (mean 79 years, IQR 69.2–83.7 vs. 71 years; IQR 62.0–81.0 in men; *p*=0.002). The majority were referred by non-palliative medicine specialists, typically from hospitals (Table 1).

**Table 1:** Patients’ characteristics on referral to palliative care

Variable	*N* (%)
Source of referral	
Hospital	135 (45.4)
General practitioner	67 (22.3)
Specialist outpatients clinic	61 (20.3)
Hospice home care	36 (12.0)
Referring physician	
Without specialization	93 (31.0)
Non-palliative medicine specialist	174 (58.0)
Palliative medicine specialist	33 (11.0)
Referral form	
Supplied with all necessary data	150 (50.0)
Supplied with some information	83 (27.7)
Containing only a diagnosis	67 (22.3)
Referral form labelled as “urgent”	84 (28.0)
Patients with disability certificate	32 (10.7)
Main diagnosis on referral	
Cancer	282 (94.0)
Digestive	90 (30.0)
Respiratory	57 (19.0)
Genitourinary	71 (23.7)
Others	64 (21.3)
Non-malignant diseases	18 (6.0)
ECOG PS on referral form	
Unreported	161 (53.7)
1	4 (1.3)
2	32 (10.7)
3	92 (30.7)
4	11 (3.6)
Stage — prognosis (GSF) on referral form	
Unreported	169 (56.3)
A – stable/years	6 (2.0)
B – unstable/months	29 (9.7)
C – progressive/weeks	85 (28.3)
D – final days	11 (3.7)

*ECOG PS* Eastern Cooperative Oncology Group performance status, *GSF* gold standards framework, *IQR* interquartile range

Only half of the referral forms were supplied with sufficient information to make the justified preliminary qualification decisions solely based on the delivered documentation. The majority lacked basic descriptions, including performance status or expected prognosis. In cases for which the required information was revealed in the referral form, approximately two-thirds were capable of only limited self-care, and being in the progressive phase of the disease, with a within-weeks life prognosis. The odds ratio of receiving a referral form supplied with all necessary data for qualification from hospital in comparison to ambulatory care was 2.676 (95% CI 1.817–4.017; *p*<0.001).

Physical symptoms or signs were given as the main reason for admission on the referral form, but in the majority of cases (53.7%), no particular reason was revealed. In the case of 139 referrals (46.3%), at least one problem was reported (median 1, IQR 1–2, range: 0–5) (Table 2).

**Table 2:** Problems reported on referrals for 139 patients

Problem	*N* (%)
Pain	50 (35.9)
Social	43 (30.9)
Dyspnoea	39 (28.0)
Nausea/vomiting	19 (13.7)
Hyperactive delirium	17 (12.2)
Oedema	13 (9.3)
Compression syndromes ^a^	11 (7.9)
Constipation/intestinal obstruction	4 (2.9)
Diarrhoea	4 (2.9)
Fever	2 (1.4)
Ascites	1 (0.7)
Advanced ulcerations	1 (0.7)

^a^Raised intracranial pressure, spinal cord compression, peripheral nerves entrapments

Social concerns were the second leading problem following pain. The number of reported problems were weakly correlated with the probability of triage as “urgent” (rho=0.25; *p*<0.001). The quality of referral forms in terms of information content improved with a higher degree of post-graduate education, reaching a sufficient level in 75.8% of palliative medicine specialists (*p*<0.001).

Twenty-eight percent of referral forms were labelled with the annotation “urgent” (Table 1). Forty percent of them did not contain any information on unresolved medical or social problems. Although more such labelled patients were triaged as “urgent cases” (47.6 vs. 31.0% without “urgent” annotation; *p*=0.009), the majority having this type of referral (52.4%) were qualified as “stable” or even (in 13.1% of cases) disqualified. In 149 patients (49.6%), it was impossible to make a justified qualification solely based on the referral form: 103 cases (34.3%) needed documentation analysis, and 46 persons (15.3%) an additional telephone interview.

Amidst those qualified, the majority (56%) were “stable” cases. Nearly one-fifth of referrals were disqualified from admission to hospice or home-care. The main reason for this was the stable phase of cancer with longer prognosis, during oncological therapy aimed at modification the natural course of the disease. One out of 10 persons referred to PC had a disability certificate, qualifying them for earlier admission, beyond the general waiting queue. Nearly two-thirds (65.6%) of these patients were qualified as “stable” cases, while 2 persons (6%) were disqualified.

Patients referred to the hospice ward (in comparison to home-care) were more often referred by doctors without specialisations than those with them (*p*<0.001), more frequently received referral forms containing necessary information to make a justified qualification decision (70.0 vs. 30.0%; *p*<0.001), were more often qualified as “urgent” cases, and less often disqualified (41.3 vs. 30.0%; *p*=0.02). In 40 (26.6%) hospice ward referrals, some social problems were signalled, in 21 (14%) as the only reason for the admission.

## Discussion

To our knowledge, this is the first study in which the quality of referrals used for palliative care admissions in Poland is described. The majority of registered patients had advanced cancer, and were referred late during the progressing disease phase; however, usually neither performance status nor prognosis were reported. This phenomenon was more evident, particularly, in the case of home-care referrals, thus, as a consequence, these patients could have been more often negatively triaged and finally disqualified. The national reimbursement system, limited mainly to cancer patients, has been the main cause of referral inequity for years. In 2021, only 26.7% of deaths were caused by neoplasms [14]; however, the prevalence of these patients within palliative care services in recent years has fluctuated around 90% [15]. Despite the evidence of increasing access for people with non-malignant illnesses worldwide, the same inequity has been visible from the beginning of the modern hospice movement in other countries [16]. This finding cannot be explained exclusively by the differences in needs between these two groups, as in the systematic review, it has been proved that there are commonalities in the prevalence of problems across cancer and non-cancer patients [17].

In more than half of the cases in our study, the only indication for specialist PC was the presence of illness contained within the strict reimbursement catalogue, which in the majority, encompasses cancers. At our hospice, to facilitate the verification process, a dedicated referral form was introduced in 1998 (Supplementary file). We found it useful for qualification, which proved the ability to predict the need of urgent palliative care with a sensitivity of 90.5% [18]. An accurate decision could be made when the presence of suffering and patients’ irreversible deterioration was observed on such form. However, in the current study, despite the long-term availability of this tool, in half of the cases, it was not implemented. Many reasons for this situation can be suspected. Some of the referring physicians may simply have ignored the significance of symptoms, not routinely carrying out screening, which is a crucial component of integration between palliative care and other medical disciplines [19]. More educational and research programs to improve this situation are still needed at all levels of health care. Apart from formal or legal provisions, our findings can be supported by the observation that referrals are influenced by complex appraisal of the situation, which goes beyond the patients’ pure prognosis or needs [20]. This assessment takes other factors into account, e.g., the needs of the referring physician, one’s emotional capacity, expertise, available time, interpretation of patients’ preferences or fears, and also assumptions about professional quality of palliative services in light of the patients’ previous input. Although it is hard to objectively detail what makes palliative needs “complex” for referral to specialist care, the percentage of reported needs on referrals in other surveys was much higher than in our study: physical needs were recorded for 76 to 91%, psychological issues for 59%, and spiritual concerns for 55% of cases [11, 21].

In spite of the fact that actual palliative care definitions are also concentrated on the earlier phases of the disease, in reality, patients continue to fear palliative care referral and associate it with cessation of active treatment and imminent death [22]. Many oncologists and hematologists, partially due to diminished trust in the competency of palliative care providers, presuppose referrals with therapeutic alliance as well as loss of hope and, in consequence, prefer to gate-keep this process, which appears to them as a daunting, time-consuming task [23, 24]. As a result, a substantial number of persons, despite evidence of a high level of unmet needs, are referred at the near-end-of-life phase [25]. What is more, delayed referrals depend not only on late initiation of serious illness conversation or prognostication made by physicians, but may also be the result of patients’ or family caregivers’ wrong perspectives of palliative care [26]. This care could incorrectly be associated with an inferior form of treatment or even approaching death, while such referral can be emotionally devastating. Finally, lack of experience of palliative care providers among patients with non-malignant diseases pose complex problems, and the insufficiency of these centres, due to excessive burden of cancer patient admissions, could be another reason for late referrals [27].

Instead of reporting patients’ needs, nearly one-third of referral forms in our survey were labelled as “urgent”, with near half of them lacking any notice of unresolved problems. This label was quite irrelevant in 50% of the situations, as the additional documentation or telephone interviews did not reveal any urgent reasons for the admission, while 1 in 8 persons could have been cared for by a general practitioner. This turns out to be similar as noted in literature on the subject, where it was found that health care providers lack knowledge and clarity regarding referrals, thus, additional education is required [28, 29].

The main suspected reason for this discrepancy between referring a physician’s convictions regarding palliative necessity and a patient’s actual needs in our study may depend on the lack of a dedicated tool available for general physicians doctors in the majority of cases, helping them in the routine screening of their patients’ needs. A number of validated screening tools are available free-of-charge, e.g., in Australia, the RUN-PC triage tool [30], with a dedicated web calculator [31], the Palliative Care Phase of Illness [32], or in Germany, the Hospice and Palliative Care Evaluation Symptoms and Problems Checklist [33]. Nonetheless, the ability of them to triage cases that are likely to have palliative needs in primary care is still limited and further research is warranted [4].

Revealing essential information regarding preliminary qualification for palliative care may also facilitate the accurate assessment of urgent indications concerning admission. It may simplify and accelerate the process, in consequence, lowering costs [18]. However, the integrative models assume a substantial role of standard care interventions, including symptom assessment, concerns regarding decision-making and advance care planning, supplemented by specialist care, when general care has failed to achieve desirable effects [34]. Routine usage of referral templates could improve the “quality” of referrals, but in reality, despite their accessibility, their feasibility in routine clinical practice could be challenging [35].

However, we noted that only in half of the cases, undisturbed triage was solely based on referral forms; therefore, in every-second patient, this qualification process had to be unnecessarily extended. This phenomenon was particularly visible when the patient was discharged from hospital. Whereas the evaluation of palliative needs is feasible from admission to hospital, also within the intensive care unit, the selection of potential candidates is done in advance. This allows changing care priorities, while facilitating timely referrals [36]. Building a collaborative team within the hospital ward and wisely choosing available hospital supportive teams is crucial, particularly, in non-cancer patients [37, 38]. An approach that allows these teams to assess treatment objectives engages the treating physician and improves the use of services [39]. Unfortunately, the actual national reimbursement system in Poland does not provide financial backing for such hospital supportive care teams, thus, in the majority of cases, leaving physicians to rely solely on their skills and knowledge.

We found that in nearly one-fifth of referrals, continuous palliative care was not necessary, as the outpatient supportive consultations seemed to be sufficient. General physicians should have basic knowledge of symptomatic management, communication skills to face and share “bad news”, and discuss advanced care plans in reaction to palliative staff deficiency [34]. They should be sufficiently equipped with both prognostic and screening tools used in routine practice. Despite the fact that primary care physicians, in most situations, should be the crucial providers of palliative care in communities, local partnerships with palliative centres are strongly recommended [12]. Generalists should cooperate with specialist outpatient palliative care at least within a consultation model, when consultants do not need to take over all aspects of care [40]. What is more, the whole health care system should also facilitate primary care development, with adequate resources and financial incentives to support education as well as collaboration within the interdisciplinary teams [41].

In our study, more than a quarter of inpatient referrals had unresolved social problems, which could be challenging when implementing hospice home-care. Nevertheless, admission of patients with predominantly social needs to specialised palliative wards can be considered as an improper use of these resources. The development of general home-care social support and long-term care facilities deserved more attention in these cases. Such types of care should be supported by palliative consultations, based on the existence of patients’ complex needs [42]. Moreover, amongst vulnerable individuals, e.g., experiencing homelessness, an additional professional health nurse navigator or case manager should coordinate healthcare transitions, facilitating timely referral [43–45]. Effective family caregiver support programmes could also improve this integration [46]. Strengthening people’s social network via the concept of compassionate communities is urgently needed as well [47].

The screening process in this study was limited to a single centre within the district town, and there were no prospective or retrospective follow-ups among the patients in terms of their problems and needs. As half of the referrals lacked the information on patients’ clinical situation, correlations presented in our study should be considered with caution. Moreover, the role of the caregivers in the referral process was not evaluated. Further multicentre studies, in which screening tools would be evaluated with adequate follow-up, are warranted.

## Conclusions

Many patients are referred to palliative care based not on one’s needs but exclusively on the presence of advanced disease. Social problems remain a significant, single indication for these referrals. General physicians need adjusted, practical tips or simple checklists in order to facilitate timely referrals and, consequently, unburden the overloaded specialist care system for better use of these resources.

## Supplementary information

ESM 1 Supplementary file: Palliative Care Referral Form (pdf). (PDF 105 kb)

## Data Availability

All relevant data are within the manuscript and its additional file. The data are available from the corresponding author on reasonable request.
