# Supplementary material for: Quality of referrals to specialist palliative care and remote patient triage — a cross-sectional study
Source: Support Care Cancer. 2023 Sep 2;31(9):551. doi: 10.1007/s00520-023-08025-6 (PMC10474992; doi:10.1007/s00520-023-08025-6)
Supplement: Supplementary file 1 — Supplementary file: Palliative Care Referral Form (pdf). (PDF 105 kb) [file 520_2023_8025_MOESM1_ESM.pdf]

Referral to Saint Lazarus Hospice

• please print legibly, according to the patient's current clinical status

Patient Last Name ______ Date of Birth ______

Patient First Name ______ Telephone ______

Address ______

Family Member for Contact ______ Relation ______

Address ______ Telephone ______

I consent to symptomatic treatment and palliative care: ______

Date and Patient's Signature (or Legal Guardian if Patient Unable)

Last Name of Referring Physician ______ First Name ______

Address ______ Telephone ______

Fax ______ E-mail ______ Signature ______

Diagnosis most-limiting Patient's Prognosis ______ **Number of hospitlizations for this reason in the last 6 months** ______

In the instance of Cancer – Location(s) of confirmed metastases? Locations of suspected metastases?

Patient Qualified for Oncological Therapy ☐ Oncological Therapy not possible; Symptom Management Recommended ☐

Remaining Significant Diagnoses

Course of Illness in the last few weeks

Current symptoms in spite of therapy

	Raised Intracranial Press. ☐	Severe Dyspnea at Rest ☐
Intensity of Pain (Moderate or Greater) ☐	Hyperactive Delirium ☐	Spinal Cord Compression ☐
Exacerbated Vomiting / Bowel Obstruction ☐	Fecal Incontinence ☐	Overwhelming Distress ☐
Superior Vena Caval Obstruction ☐	Advanced Edema ☐	Other: ☐

Deterioration of Clinical Status: within days ☐ weeks ☐ months ☐ years ☐

Degree of Ability according to Palliative Performance Scale: (please circle)

		30% Completely dependent, bed bound
80% Normal activity with effort	50% Regular assistence, sitting-bed bound	20% Able only to minimal intake
60% Occasional assistance required	40% Mostly dependent, bed-bound	10% Mouth care only

In those without an oncological diagnosis, please provide current biochemical lab values and assess the state of decubitus ulcers:

Leukocytes ☐	Creatinine ☐	Serum Albumin ☐	Number of Ulcers ☐	No of infected ☐
Hemoglobin ☐	Serum Urea ☐	C-reactive protein ☐	Stage of most advanced Decubitus Ulcer (I-IV) ☐	

Current Therapy (International/Generic Names, doses)

Factors hindering the patient's stay at home, comments

Does the Patient possess any insurance which may assist in funding care? Yes ☐ No ☐
